# Self-reported mental health among individuals with mental disorders during the COVID-19 pandemic

**DOI:** 10.1192/j.eurpsy.2021.126

**Published:** 2021-08-13

**Authors:** S. Østergaard, P. Kølbæk, M. Speed, O. Jefsen

**Affiliations:** 1 Department Of Affective Disorders, Aarhus University Psychiatric Hospital, Aarhus, Denmark; 2 Psychosis Research Unit, Aarhus University Hospital - Psychiatry, Aarhus N, Denmark; 3 Department Of Affective Disorders, Aarhus University Hospital - Psychiatry, Aarhus N, Denmark; 4 Department Of Affective Disorders, Aarhus University Hospital, Aarhus, Denmark

**Keywords:** COVID-19, psychopathology, pandemic, mental health

## Abstract

**Background:**

Individuals with mental illness may be particularly vulnerable to the negative impact that the coronavirus disease 2019 (COVID-19) pandemic seems to have on mental health. Most prior studies on this topic are however limited by non-random sampling, lack of information on non-respondents, and self-reported diagnoses. Here, we aimed at overcoming these limitations by means of random sampling in a population of clinically diagnosed patients, acquisition of clinical and socio-demographic data on non-respondents, and weighting of results informed by attrition.

**Methods:**

We conducted a cross-sectional questionnaire-based online survey inviting six-thousand randomly drawn patients from the psychiatric services of the Central Denmark Region. They survey data were merged with sociodemographic- and clinical data from medical records on all invitees, which enabled analysis of attrition and weighting of results. The questionnaire included the 18-item Brief Symptom Inventory (BSI-18), the 5-item World Health Organization Well-Being Index (WHO-5), and 14 questions evaluating the perceived severity of symptoms during the four-week nationwide lockdown of Denmark in March/April 2020 – using the pre-pandemic period as reference. Reasons for worsening or improvement in mental health during lockdown were also reported.

**Results:**

The preliminary results are as follows: The response rate was ≈20%. Approximately half the respondents reported that their mental health had deteriorated during lockdown, while the other half reported either no change (≈33%) or improvement (≈16%). The most commonly reported reasons for deterioration in mental health were disruption of routines and loneliness.

**Conclusion:**

The final results will be shown at the conference.

**Disclosure:**

No significant relationships.

